# *DUSP5* is methylated in CIMP-high colorectal cancer but is not a major regulator of intestinal cell proliferation and tumorigenesis

**DOI:** 10.1038/s41598-018-20176-9

**Published:** 2018-01-29

**Authors:** Lars Tögel, Rebecca Nightingale, Rui Wu, Anderly C. Chüeh, Sheren Al-Obaidi, Ian Luk, Mercedes Dávalos-Salas, Fiona Chionh, Carmel Murone, Daniel D. Buchanan, Zac Chatterton, Oliver M. Sieber, Diego Arango, Niall C. Tebbutt, David Williams, Amardeep S. Dhillon, John M. Mariadason

**Affiliations:** 1 Olivia Newton-John Cancer Research Institute, Melbourne, Australia, La Trobe University School of Cancer Medicine, Melbourne, Australia; 2 Ludwig Institute for Cancer Research, Melbourne, Australia; 3 Colorectal Oncogenomics Group, Genetic Epidemiology Laboratory, Department of Pathology, The University of Melbourne, Parkville, Melbourne, Australia; 4 Systems Biology and Personalised Medicine Division, The Walter and Eliza Hall Institute of Medical Research, Melbourne, Australia; 5 Group of Biomedical Research in Digestive Tract Tumours, CIBBIM-Nanomedicine, Vall d’Hebron Research Institute (VHIR), Universitat Autonoma de Barcelona, Barcelona, Spain

**Keywords:** Colorectal cancer, Oncology

## Abstract

The ERK signalling pathway regulates key cell fate decisions in the intestinal epithelium and is frequently dysregulated in colorectal cancers (CRCs). Variations in the dynamics of ERK activation can induce different biological outcomes and are regulated by multiple mechanisms, including activation of negative feedback loops involving transcriptional induction of dual-specificity phosphatases (DUSPs). We have found that the nuclear ERK-selective phosphatase DUSP5 is downregulated in colorectal tumours and cell lines, as previously observed in gastric and prostate cancer. The *DUSP5* promoter is methylated in a subset of CRC cell lines and primary tumours, particularly those with a CpG island methylator phenotype (CIMP). However, this epigenetic change alone could not account for reduced DUSP5 expression in CRC cells. Functionally, DUSP5 depletion failed to alter ERK signalling or proliferation in CRC cell lines, and its transgenic overexpression in the mouse intestine had minimal impact on normal intestinal homeostasis or tumour development. Our results suggest that DUSP5 plays a limited role in regulating ERK signalling associated with the growth of colorectal tumours, but that methylation the *DUSP5* gene promoter can serve as an additional means of identifying CIMP-high colorectal cancers.

## Introduction

The ERK signalling pathway couples extracellular and oncogenic signals with molecular networks that control cell proliferation, differentiation, survival, and motility^1,2^. Activation of the pathway occurs when cell surface receptors such as the EGF receptor (EGFR) induce GTP-loading of RAS, which initiates sequential activation of RAF, MEK and ERK protein kinases. ERKs can phosphorylate a multitude of cytoplasmic and nuclear proteins, the combination of which depends on the duration, magnitude, and site of ERK activation^3^.

A key mechanism whereby cells control the dynamics of ERK signalling is by inducing negative feedback loops. This involves either direct ERK-mediated phosphorylation of RAF proteins, or transcriptional induction of upstream signalling inhibitors (eg. Sprouty2/4) and/or dual-specificity phosphatases (DUSPs) that directly target ERK^4^. These systems operate in a cell-specific manner and act at multiple steps of the pathway to maintain signalling flux at levels optimal for normal cell function.

DUSP proteins dephosphorylate tyrosine and serine/threonine residues required for activation of ERK, JNK and p38 mitogen-activated protein kinases (MAPKs)^5,6^. They contain a C-terminal catalytic domain, a N-terminal domain controlling subcellular localization, and a kinase interacting motif. DUSPs are broadly classified into 3 groups based on their localization: group I DUSPs (DUSPs 1, 2, 4 and 5) are nuclear, group II DUSPs (DUSP 6, 7 and 9) are cytoplasmic, and group III DUSPs (DUSPs 8, 10 and 16) exist in both the nucleus and cytoplasm. Functionally, individual DUSPs often exhibit preference for specific MAPK substrates, with DUSPs 5, 6, and 7 being ERK-selective while DUSP9 prefers ERK over the p38 and JNK MAPKs.

DUSP5 is a nuclear ERK1/2-selective phosphatase induced by ERK signalling in mammalian cells^7,8^. It also controls ERK pathway activity by anchoring inactive ERK proteins in the nucleus, preventing MEK-mediated reactivation of ERK in the cytoplasm^8^. In mice, DUSP5 deficiency in the epidermis increases sensitivity to *H-Ras*-driven papilloma formation in a carcinogen-induced model of skin cancer^9^.

Consistent with this putative tumour suppressor role, DUSP5 expression is downregulated in prostate and gastric cancers where loss of expression is associated with poor prognosis^10–12^. In gastric cancer, DUSP5 downregulation was reported to correlate with promoter CpG island methylation, and its re-expression in gastric cancer cell lines reduced nuclear p-ERK levels and cell growth^12^.

Downregulation of DUSP5 was also recently reported in colorectal cancer (CRC), and shown to be associated with worse outcome^10^. However, the mechanisms underlying DUSP5 loss in this disease, and whether its loss contributes to aberrant ERK signalling or the initiation and/or progression colorectal cancers has not been established. In this study, we confirm that *DUSP5* is significantly downregulated in CRC. Furthermore, we show that the *DUSP5* promoter is methylated in a subset of CRCs, specifically those harboring the CpG island methylator phenotype (CIMP). However, promoter methylation alone did not correlate with altered *DUSP5* expression in CRC cell lines or primary tumours, suggesting multiple mechanisms contribute to DUSP5 downregulation in this disease. Unexpectedly, knockdown of endogenous DUSP5 in CRC cell lines did not affect ERK signaling or proliferation, while transgenic overexpression of DUSP5 in the mouse intestine had little impact on normal intestinal homeostasis or tumorigenesis.

## Results

### *DUSP5* is downregulated in colorectal cancers (CRCs)

**Figure 1:**
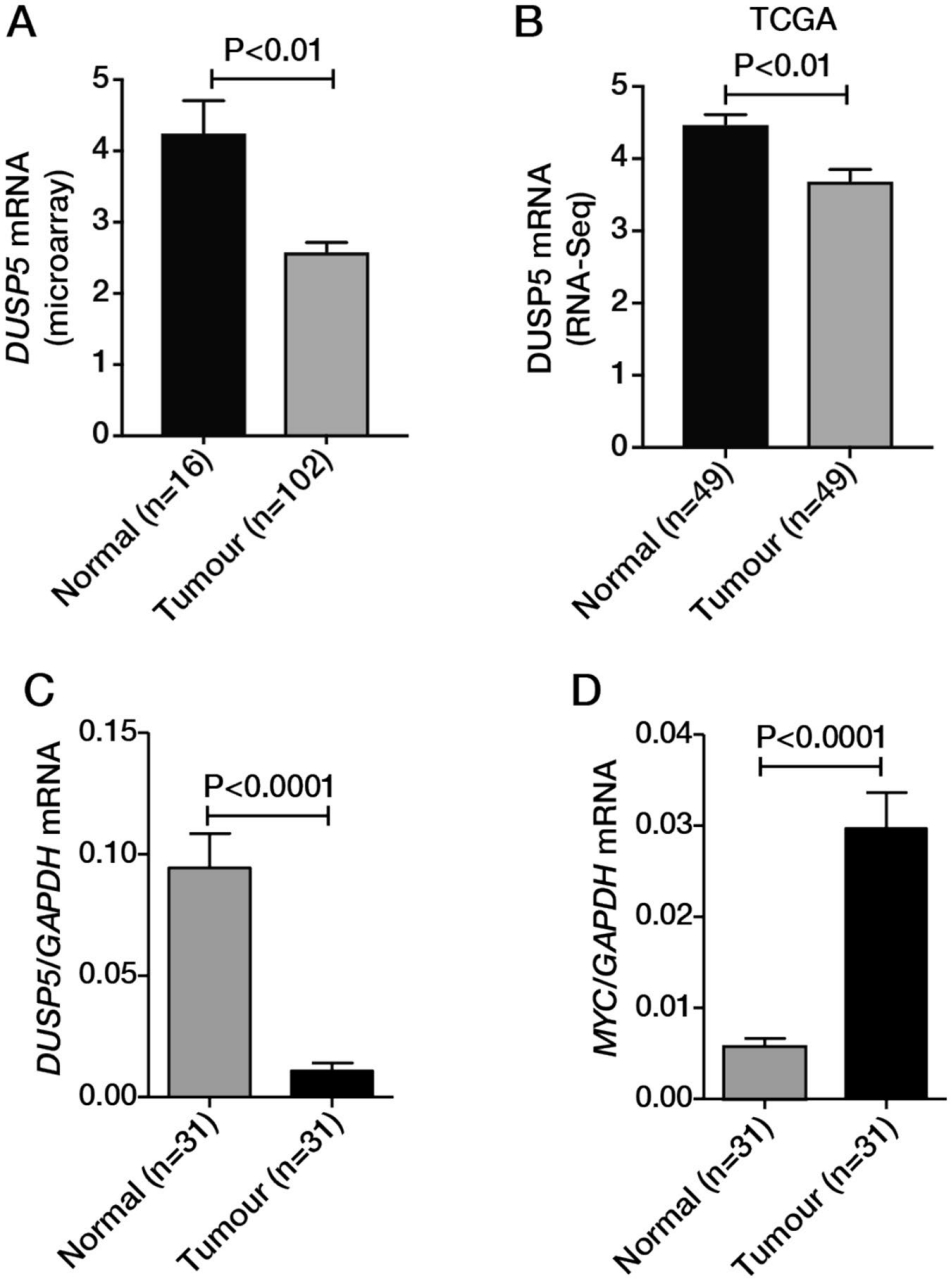
Downregulation of *DUSP5* mRNA expression in CRC. *DUSP5* mRNA expression in normal colonic mucosa and colorectal tumours determined by analysis of (**A**) an in-house Affymetrix microarray dataset and (**B**) RNA-Seq data available from the TCGA. (**C**) *DUSP5* and (**D**) *MYC* mRNA expression in colon tumour/normal pairs normalised to *GAPDH* levels.

*DUSP5* is a putative tumour suppressor gene that is aberrantly expressed in several cancers. To determine if the expression of *DUSP5* is altered in CRC, we interrogated an in-house microarray dataset comprising 102 human CRCs and normal colonic tissue from 16 individuals (Fig. 1A), and RNA-seq data from the TCGA of 49 CRCs and matched adjacent normal tissue (Fig. 1B). Both datasets revealed significantly lower *DUSP5* expression in CRC compared to normal tissue. This finding was further verified by qPCR analysis using an independent patient cohort consisting of 31 matched pairs of CRCs and normal colonic tissue (Fig. 1C). The tumour and normal colonic origins of the specimens is illustrated by higher levels of *MYC* in the tumour tissue (Fig. 1D).

### DUSP5 is induced by ERK signalling in CRC cell lines

**Figure 2:**
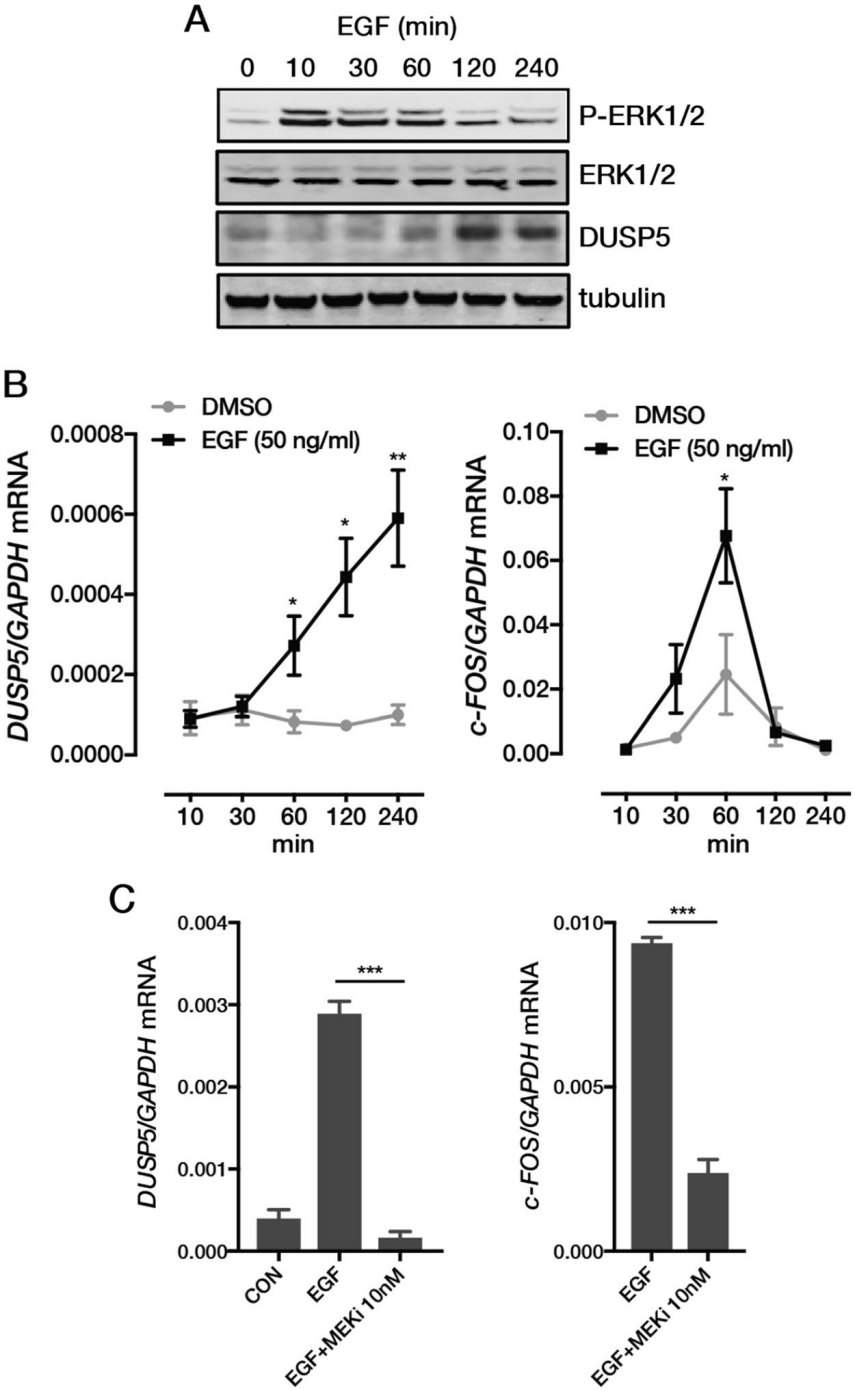
Induction of *DUSP5* by EGFR-ERK signalling in CRC cells. Time-course showing EGF-mediated induction of (**A**) p-ERK1/2 and DUSP5 protein expression, and (**B**) *DUSP5* and *c-FOS* gene expression in LIM1215 cells. (**C**) Induction of *DUSP5* and *c-FOS* mRNA by EGF requires MEK/ERK signalling. LIM1215 cells were stimulated with EGF (50 ng/ml) for 24 h in the absence or presence of the MEK inhibitor (MEKi) trametinib. ^*^P < 0.05, ^**^P < 0.005, ^*^P < 0.0005.

*DUSP5* expression is induced by ERK signalling in several cell types^6,7^. To examine the dynamics of *DUSP5* induction in CRC cells, we treated the *KRAS*/*BRAF* wild-type CRC cell line LIM1215 with epidermal growth factor (EGF), which induced a transient and robust increase in ERK phosphorylation (Fig. 2A). EGF also invoked a significant increase in DUSP5 protein levels but in a delayed and more prolonged manner compared to ERK phosphorylation, consistent with its role as a negative feedback regulator of this pathway. Examination of these effects at the transcriptional level demonstrated that EGF also induced a delayed but sustained induction of *DUSP5*, in contrast to the transient induction of the well-established ERK target, *FOS* (Fig. 2B). EGF induction of *DUSP5* and *FOS* were also strongly attenuated by a MEK inhibitor (trametinib), demonstrating that *DUSP5* is a *bone fide* target of ERK signaling in *KRAS*/*BRAF* wild-type CRC cells (Fig. 2C).

### The *DUSP5* promoter is methylated in a subset of CRCs

**Figure 3:**
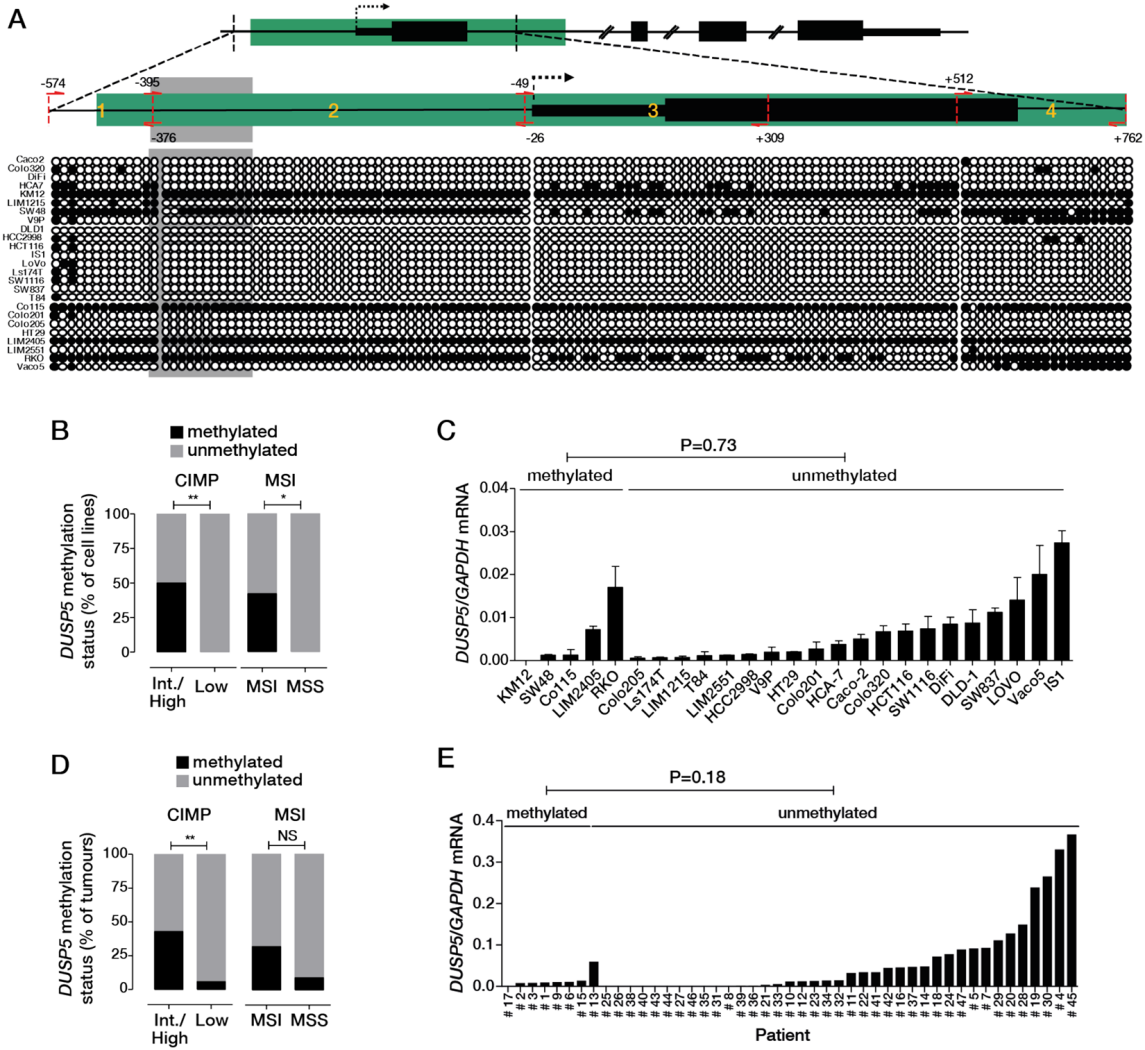
*DUSP5* promoter methylation in CRC cell lines and tumours. (**A**) Schematic of the *DUSP5* gene showing the location of the CpG island (highlighted in green). The transcription start site is indicated by dotted arrow. Zoomed image shows the location of the 4 amplicons investigated for methylation change by bisulphite sequencing. Bottom panel shows the methylation status of specific CpGs (circles) within each amplicon in a panel of 25 CRC cell lines. Open circles indicate non-methylated and closed circles indicate methylated CpGs. *KRAS*/*BRAF* wild-type cell lines are shown at the top of the panel (Caco2-V9P), *KRAS* mutant lines in the middle DLD1-T84) and *BRAF* mutant lines at the bottom (Co115-Vaco5). (**B**) Classification of the *DUSP5* promoter methylation status of the 25 CRC cell lines relative to CIMP and MSI status. (**C**) *DUSP5* mRNA expression in the 25 CRC cell lines determined by qPCR. (**D**) Classification of the *DUSP5* promoter methylation status of 47 CRC’s according to CIMP and MSI status. (**E**) *DUSP5* mRNA expression in the 47 human CRC’s separated according to *DUSP5* promoter methylation status. ^*^P < 0.05, ^**^P < 0.005.

Downregulation of *DUSP5* expression in gastric cancer is associated with hypermethylation of a CpG island in its promoter^12^. To determine if this epigenetic mechanism contributes to downregulation of *DUSP5* in CRCs, we used bisulphite conversion and sequencing to interrogate 4 contiguous regions spanning the *DUSP5* proximal promoter, exon 1, and the first part of intron 1, in a panel of 25 CRC cell lines (Fig. 3A). The 4 regions were found to be methylated in 5 cell lines (KM12, SW48, Co115, LIM2405 and RKO, Fig. 2B), all of which are microsatellite unstable (P = 0.046, Fisher’s Exact Test) and feature the CpG island methylator high phenotype (CIMP-H) (P = 0.009) (Fig. 3B). However, *DUSP5* promoter methylation was not significantly associated with reduced *DUSP5* mRNA levels across the cell line panel, with some heavily methylated cell lines (RKO) expressing high levels of *DUSP5* (Fig. 3C).

To assess *DUSP5* promoter methylation in primary CRCs, we developed a high-resolution melt (HRM) assay applicable to DNA extracted from formalin-fixed paraffin-embedded (FFPE) tissue samples. The assay interrogates a 51 base pair fragment harboring 9 CpG dinucleotides (Fig. 3A, grey shaded region) within the proximal *DUSP5* promoter. After validating its efficacy in discriminating *DUSP5* promoter methylation states in cell lines (Fig. S1), we used the HRM assay to evaluate DUSP5 promoter methylation status in 47 CRCs, which included 14 with a CIMP-Intermediate/High (CIMP-I/H) phenotype. *DUSP5* promoter methylation was identified in 8/47 cases (17%). As observed in CRC cell lines, the methylation was more frequent in CIMP I/H tumours (6/14) compared to CIMP-Low (2/33) cases (<0.005, Fisher’s exact test) (Fig. 3D), however, was not associated with reduced *DUSP5* mRNA levels (Fig. 3E, p = 0.189, unpaired t-test). Collectively, these data indicate that *DUSP5* promoter methylation represents a novel marker of CIMP status in CRC but is not the predominant mechanism of *DUSP5* repression in these tumours.

### DUSP5 is not a major regulator of ERK signalling in CRC cells

**Figure 4:**
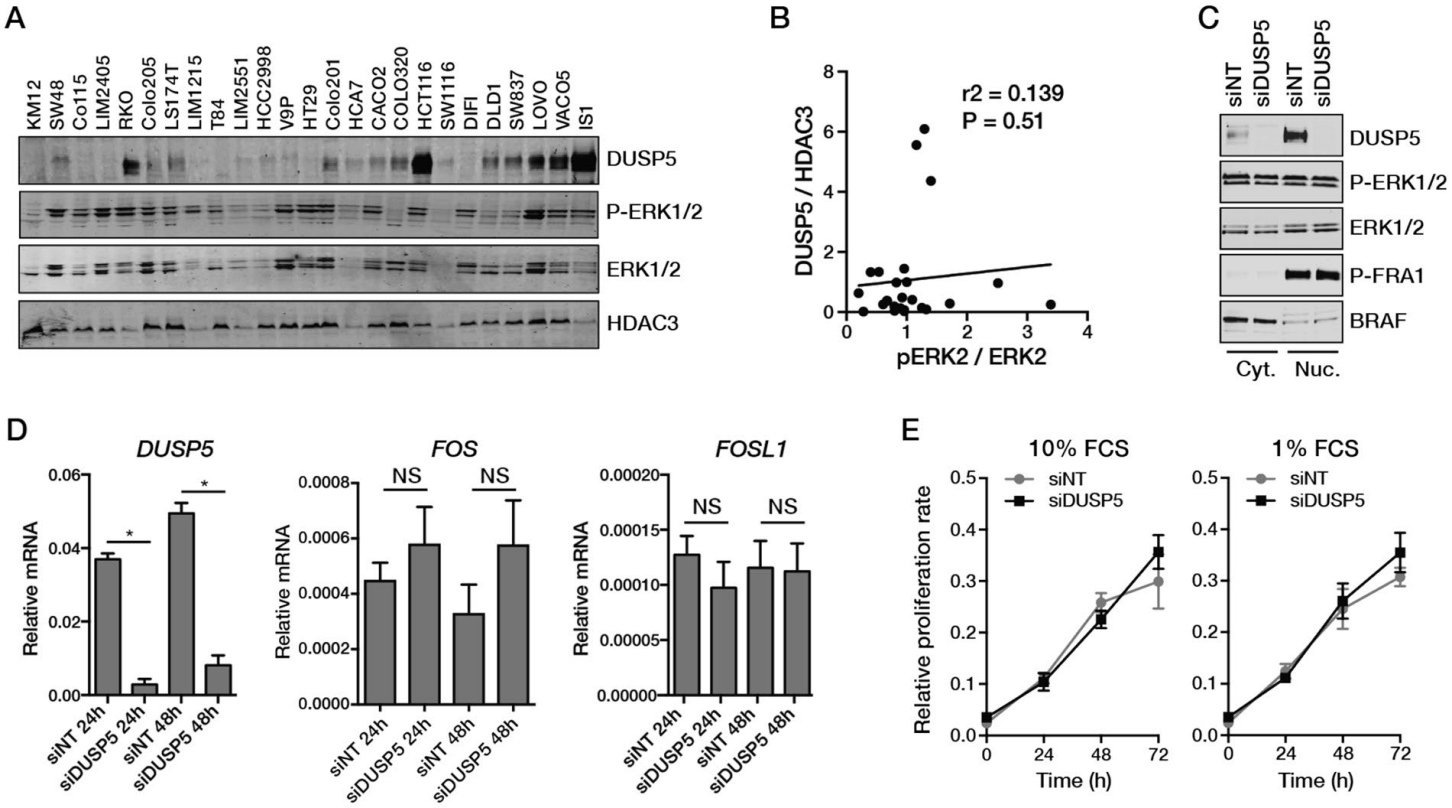
DUSP5 regulation of basal ERK signaling in CRC cells. (**A**) Relationship between DUSP5 expression and levels of phosphorylated and total ERK1/2 in nuclear extracts of CRC cell lines. HDAC3 was used as loading control. (**B**) Analysis of the correlation between DUSP5 expression normalized to HDAC3 and the p-ERK2/ERK2 ratio following densitometric analysis of the data shown in (**A**). (**C**) Effect of DUSP5 knockdown in RKO cells on cytoplasmic and nuclear levels of phosphorylated and total ERK1/2, and ERK-mediated phosphorylation of the transcription factor FRA1 (*FOSL1*). BRAF was used as cytoplasmic marker. (**D**) Effect of DUSP5 knockdown on expression of the ERK-regulated genes *FOSL1* and *FOS* in RKO cells. (**E**) Effect of DUSP5 knockdown on proliferation of RKO cells grown under normal (10% FCS) or low (1% FCS) serum conditions. ^*^P < 0.05.

The major functions of DUSP5 described to date are to dephosphorylate nuclear ERK1/2 proteins and to sequester their inactive forms in the nucleus^6,8^. We therefore assesed whether basal DUSP5 expression correlates with the levels of nuclear p-ERK in a panel of 25 CRC cell lines. Basal levels of DUSP5 varied significantly among the cell lines but displayed no clear relationship with the ratio of phosphorylated to total ERK1/2 in the nucleus (Fig. 4A,B).

DUSP5 levels have been reported to be elevated in some cancers, particularly in the context of *BRAF* mutations^13,14^. Studies in DUSP5-deficient MEFs suggest that these high levels of DUSP5 serve to dampen ERK pathway hyperactivation to facilitate transformation by mutant BRAF^15^. To test if endogenous DUSP5 plays a similar role in CRC cells, we used the *BRAF* mutant RKO cell line, which expresses high levels of DUSP5 (Fig. 4A) and is dependent on ERK signalling for proliferation (Fig. S2). DUSP5 knockdown in RKO cells did not alter nuclear or cytoplasmic phospho-ERK1/2 levels or phosphorylation of the nuclear ERK substrate FRA1 (Fig. 4C). DUSP5 knockdown also had no effect on ERK-dependent transcriptional induction of *FOS* or *FOSL1* ^16,17^ (Fig. 4D). Finally, DUSP5 depletion did not affect proliferation of RKO cells grown under standard or low-serum conditions (Fig. 4E). These results indicate that endogenous DUSP5 plays a limited role in regulating basal levels of ERK signalling in *BRAF* mutant CRC cells.

**Figure 5:**
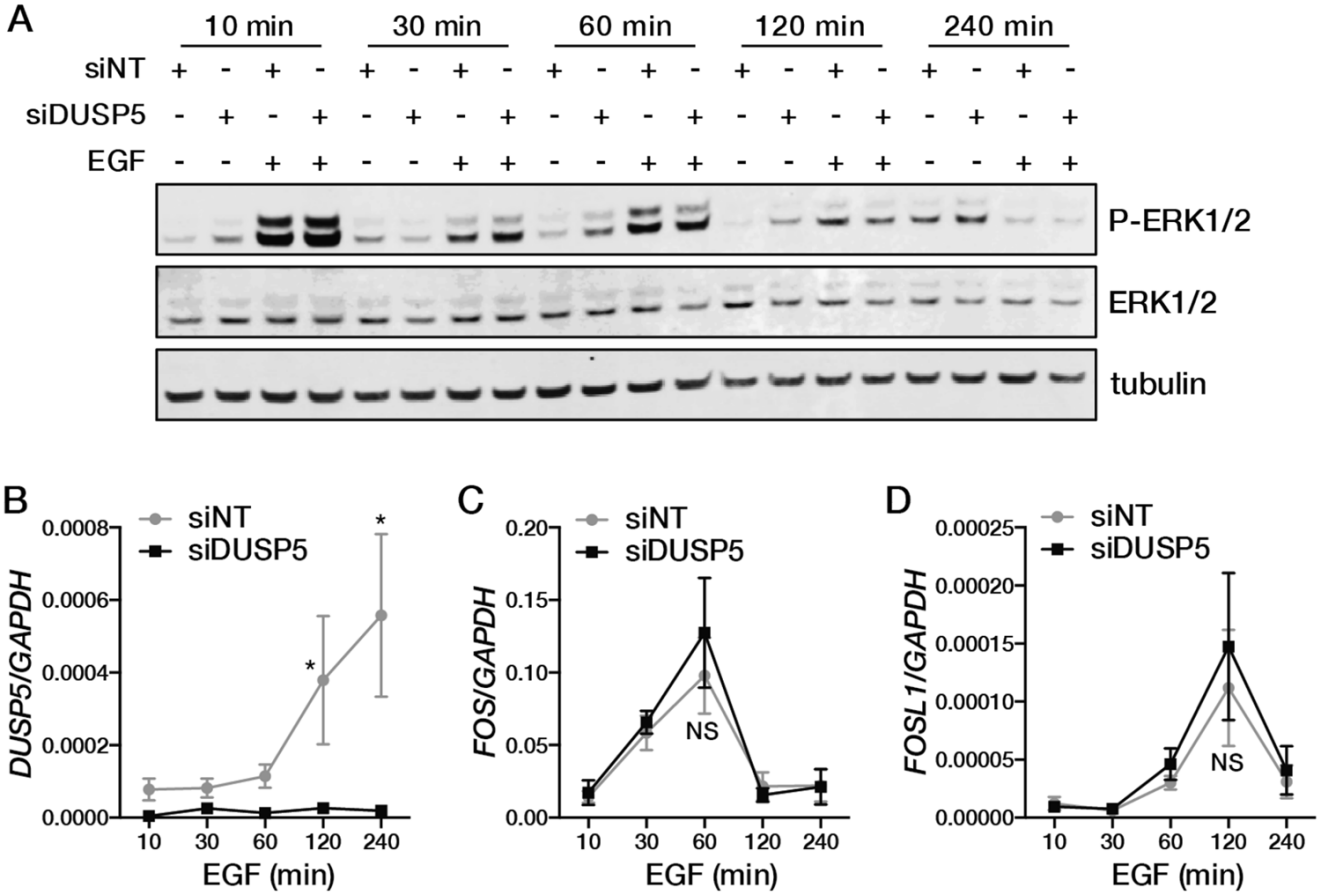
DUSP5 regulation of EGF-induced ERK signalling in CRC cells. Effect of DUSP5 knockdown on induction of (**A**) ERK1/2 phosphorylation, and mRNA levels of (**B**) *DUSP5*, (**C**) *FOS*, and (**D**) *FOSL1* following treatment of LIM1215 cell with EGF (50 ng/ml).

Next, we examined if induction of DUSP5 following EGF stimulation regulates the magnitude or duration of EGF-induced ERK signalling in CRC cells. Knockdown of *DUSP5* expression in *KRAS*/*BRAF* wild-type LIM1215 cells had no effect on the dynamics of EGF-induced ERK phosphorylation or transcriptional induction of *FOS* or *FOSL1* (Fig. 5A–C). Collectively, our data suggest that although DUSP5 is induced upon ERK pathway activation, it is not a major regulator of ERK signalling in CRC cells.

### DUSP5 overexpression in the mouse intestinal epithelium does not alter ERK signalling, intestinal homeostasis or adenoma formation

**Figure 6:**
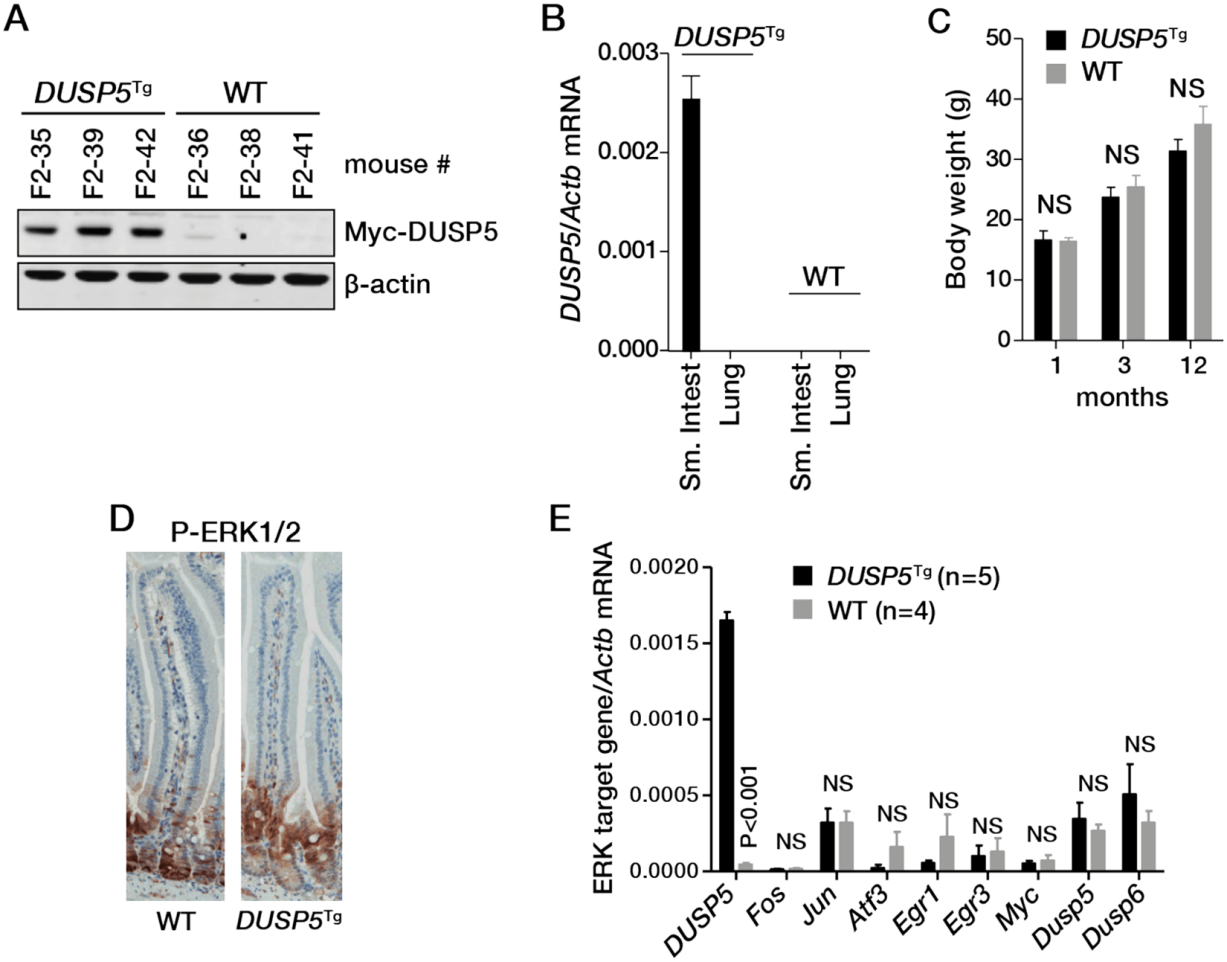
Impact of *DUSP5* overexpression on ERK signaling in the mouse intestine. (**A**) Expression of human DUSP5 protein in the intestinal epithelium detected by blotting for the Myc-tag. (**B**) Expression of human *DUSP5* mRNA specifically in the intestinal epithelium of transgenic mice. (**C**) Body weight of wild-type and *Vil*-*DUSP5* ^Tg^ mice determined over time. Effects of DUSP5 overexpression on the (**D**) intensity and pattern of pERK1/2 staining and (**E**) expression ERK signalling-regulated genes in the intestinal epithelium. Three mice of each genotype were analysed.

To complement our cell line studies and test the growth suppressive effects of DUSP5 *in vivo*, we generated a mouse strain in which expression of a human Myc-tagged DUSP5 transgene is driven by the intestinal-specific villin promoter (*Vil1*-*DUSP5* ^Tg^). The functionality of Myc-tagged human DUSP5 has previously been demonstrated in mouse embryo fibroblasts and *in vivo* studies involving transgene expression in the lymphoid compartment of mice^15,18^. Overexpression of Myc-DUSP5 specifically in the small intestine was confirmed at the mRNA and protein level by detection of human *DUSP5* mRNA and the Myc-tag, respectively (Fig. 6A,B). *Vil1*-*DUSP5* ^Tg^ mice were viable, fertile and lacked a discernible phenotype, including no difference in body weight at 1, 3 or 12 months (Fig. 6C). We were unable to detect overexpression of the Myc-DUSP5 protein in the colonic epithelium, in agreement with previous reports that transgene expression driven by the villin promoter is more robust in the mouse small intestine^19^. All subsequent studies were therefore performed on this tissue.

To determine if DUSP5 overexpression alters ERK signalling in this tissue, we assessed p-ERK1/2 levels by immunohistochemistry. No difference in the intensity and pattern of p-ERK1/2 staining was evident in the crypt-villus axis between WT and *Vil1*-*DUSP5* ^Tg^ mice (Fig. 6D). Similarly, analysis of intestinal epithelial cells isolated from WT and *Vil1*-*DUSP5* ^Tg^ mice revealed no difference in expression of the ERK signalling-regulated genes *Fos*, *Jun*, *Atf3*, *Egr1*, *Egr3*, *Myc*, *Dusp5* and *Dusp6* (Fig. 6E).

**Figure 7:**
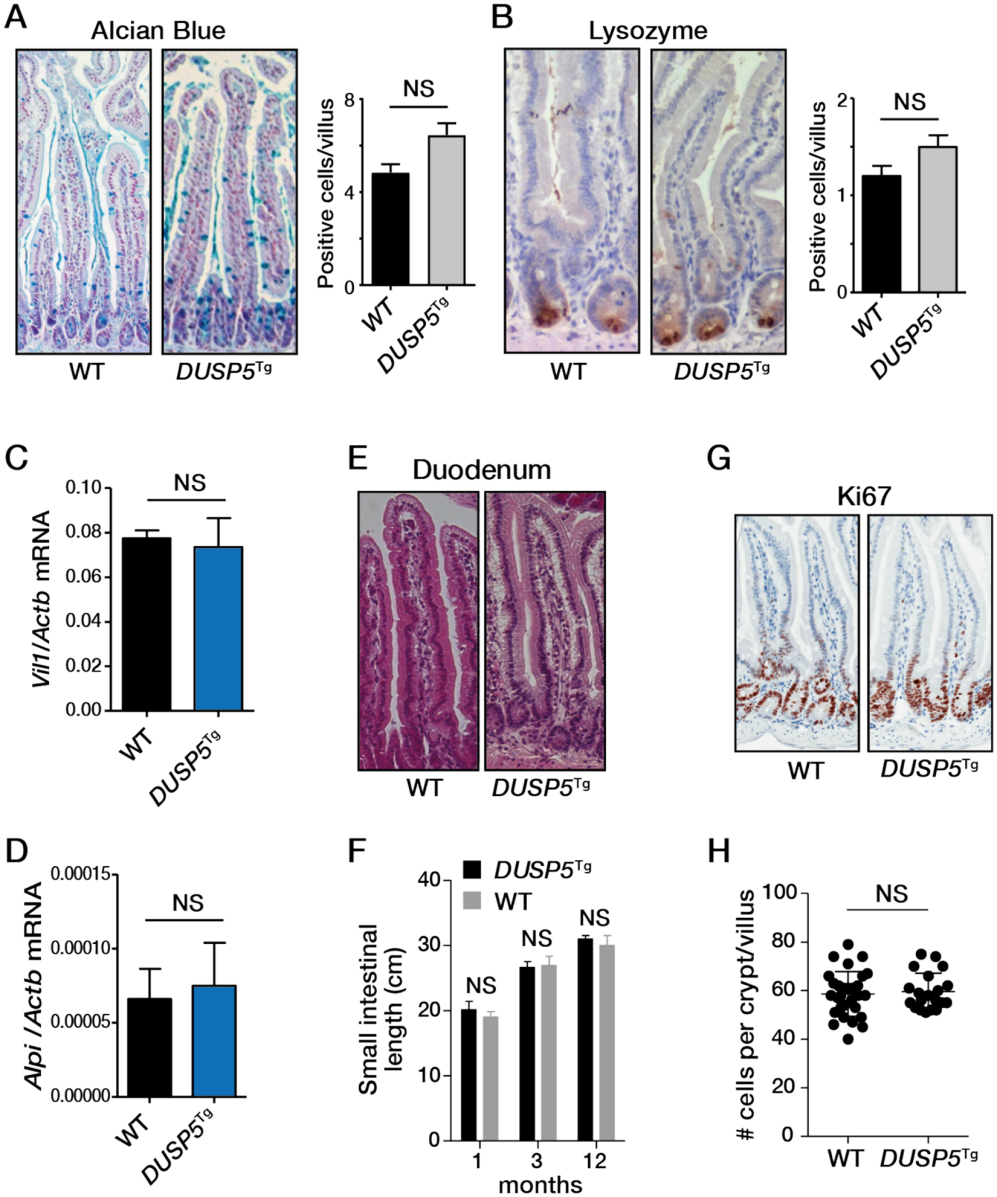
Impact of DUSP5 overexpression on intestinal phenotype. Effect of DUSP5 overexpression on cell differentiation as determined by staining for (**A**) Goblet cell (Alcian Blue) and (**B**) Paneth cell (lysozyme) markers, and measuring mRNA levels of (**C**) villin (*Vil1*) and (**D**) alkaline phosphatase (*Alpi*). Effect of DUSP5 overexpression on (**E,F**) small intestinal length, (**G**) cell proliferation and (**H**) crypt cell number (10 crypts per mouse; 3 mice per genotype).

ERK signalling plays an important role in regulating normal cell proliferation and maturation in the mouse intestine^20^, particularly differentiation along the secretory cell lineage^21^. However, no significant differences in numbers of goblet (based on Alcian Blue staining) or Paneth cells (based on staining for lysozyme) were detected between WT and *Vil1*-*DUSP5* ^Tg^ mice (Fig. 7A,B). DUSP5 overexpression also had no effect on expression of the absorptive lineage markers, villin (*Vil1*) and alkaline phosphatase (*Alpi*) (Fig. 7C,D). Similarly, no differences in length of the crypt-villus axis (Fig. 7E), length of the small intestine (Fig. 7F), rate of cell proliferation in the small intestine (Fig. 7G), or cell number along the crypt-villus axis (Fig. 7H) was detected between WT and *Vil1*-*DUSP5* ^Tg^ mice (Fig. 7E–H). Collectively, these findings indicate that DUSP5 overexpression has minimal effects on ERK signalling and cell homeostasis in the normal intestinal epithelium.

**Figure 8:**
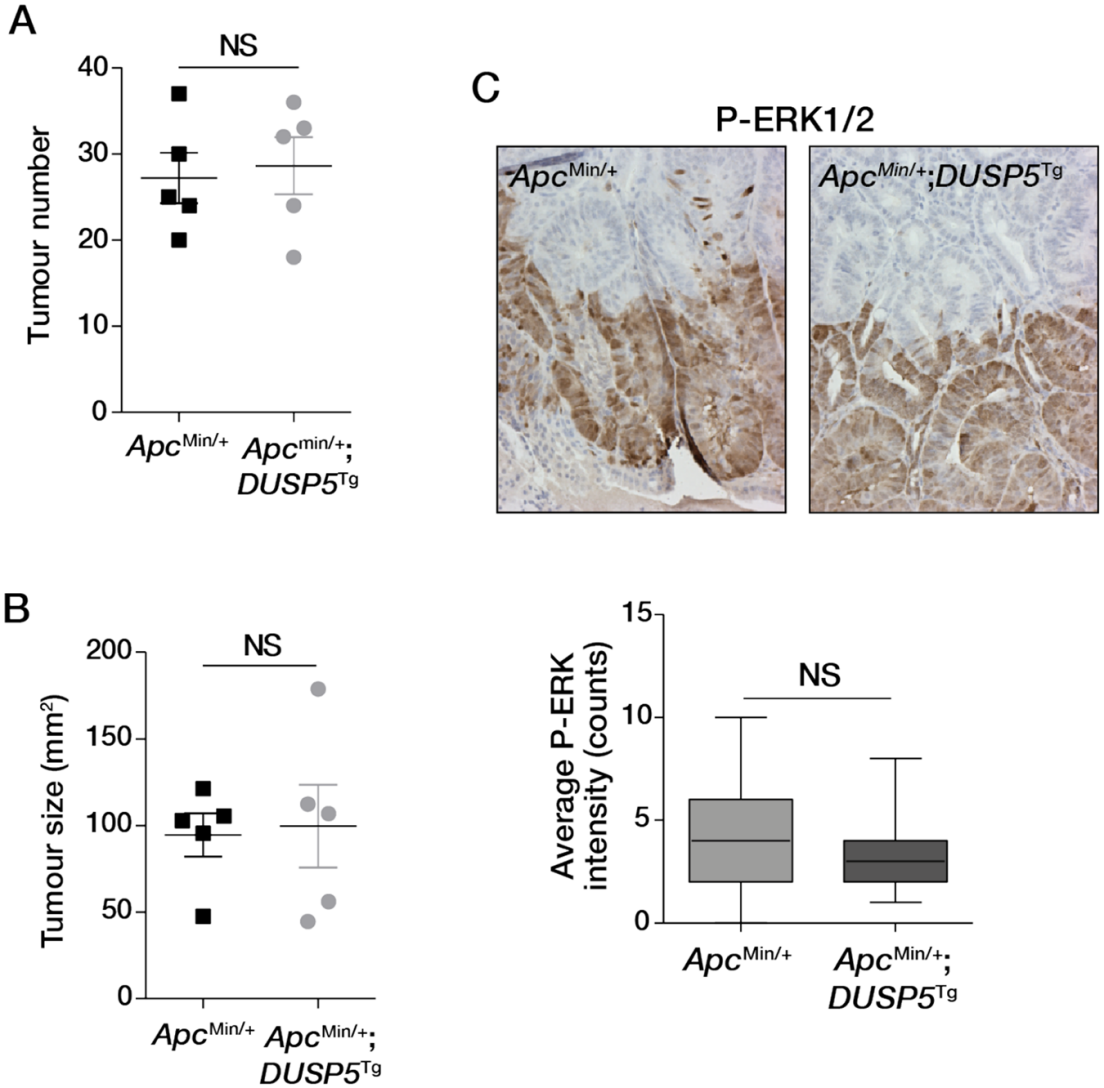
Impact of DUSP5 overexpression on intestinal adenoma formation. Effect of DUSP5 overexpression on the (**A**) number and (**B**) size of intestinal tumours that develop upon *Apc* loss. (**C**) DUSP5 overexpression does not alter the pattern or intensity of pERK1/2 staining in intestinal adenomas. Five mice of each genotype were analysed.

Finally, previous work has shown that *DUSP5* exhibits tumour suppressive activity in a mouse model of carcinogen-induced skin tumorigenesis^9^. To determine the tumour suppressive effects of DUSP5 in the intestinal epithelium *in vivo*, we crossed *Vil1*-*DUSP5* ^Tg^ with *Apc* ^Min/+^ mice. Adenoma formation in *Apc* ^Min/+^ mice requires ERK signalling^22^ and this model is widely used to investigate mechanisms regulating *Apc* mutation-initiated intestinal adenoma formation^23^. *Apc* ^Min/+^ mice developed the expected number of intestinal adenomas at 4 months of age, which was not affected by *DUSP5* overexpression (Fig. 8A). Similarly, *DUSP5* overexpression had no impact on tumour size (Fig. 8B) or the pattern and intensity of pERK staining in the adenomas (Fig. 8C). These findings indicate that DUSP5 overexpression does not exert tumour suppressive activity in a mouse model of intestinal tumorigenesis.

## Discussion

In this study we provide clear evidence of DUSP5 downregulation in CRCs compared to normal colonic tissue. We show that the *DUSP5* promoter is methylated in both CRC cell lines and patient tumours, but in contrast to previous findings in gatric cancer, our data indicate that this epigenetic mechanism alone is insufficient to account for downregulation of *DUSP5*. Notably, *DUSP5* promoter methylation occurs predominantly in CIMP-high CRCs, where multiple genes and loci are coordinately methylated^24^. Thus, *DUSP5* may be a novel addition to the panel of genes preferentially methylated in this tumour subset.

While the lack of correlation between *DUSP5* promoter methylation and gene expression was unexpected, this observation is consistent with genomic analyses indicating that the majority of genes methylated in CRC are not altered in expression^25^. In fact, we found that some cell lines with extensive methylation of the DUSP5 promoter, such as RKO, feature high DUSP5 expression, likely due to strong ERK pathway activation driven by mutant *BRAF*. DUSP5 overexpression has been documented in several tumour types and cell lines harboring *BRAF* mutations^13,14^. Recent studies using DUSP5-deficient MEFs indicate that the elevated DUSP5 levels function to prevent senescence by limiting ERK pathway activation to a level that promotes cell proliferation and transformation^15^. In contrast to these data, we found that knockdown of endogenous *DUSP5* did not alter proliferation, p-ERK levels or phosphorylation of the nuclear-localised ERK substrate FRA1 in *BRAF* mutant CRC cells. Furthermore, transgenic overexpression of *DUSP5* in the mouse intestinal epithelium did not alter basal p-ERK levels or ERK-regulated gene expression, cell differentiation, proliferation or adenoma formation, processes that require activation of this pathway^20,22^. Importantly, our findings are consistent with the lack of any discernible phenotype of *DUSP5* knockout mice that has been previously reported, other than the differential response to DMBA challenge^9^.

Our findings raise the possibility that other DUSP family members or negative feedback regulators, play more dominant roles in regulating the ERK pathway on the normal intestinal epithelium and in CRC. Indeed, previous studies have identified an important role for DUSP4 in regulating nuclear ERK phosphorylation in intestinal epithelial cells and in CRC cell lines^26^. It was also reported that DUSP6 induction by p53 reduces global phospho-ERK levels in HCT116 CRC cells^27^.

Collectively, our data indicate that DUSP5 is not a major negative regulator of ERK signalling in the intestinal epithelium and has limited tumour suppressive activity in CRC. Promoter methylation of *DUSP5* in these cancers may be a passenger event, which differs from its role in gastric cancer cells. It however remains possible that DUSP5 may regulate the functions of the pathway in contexts other than those examined in the present study. Notably, DUSP5 downregulation in CRC has been shown to be adversely prognostic in advanced but not early stage disease, where it is associated with metastasis and markers of epithelial-mesenchymal transition^10^. Further studies are thus needed to determine if DUSP5 contributes to regulation of the ERK pathway in more advanced stages of colorectal tumorigenesis.

## Methods

### Cell lines and treatments

Colorectal cancer cell lines were maintained at 37 °C and 5% CO_2_ in Dulbecco’s Modified Eagle’s Minimal Essential Medium Nutrient Mixture F-12 (DMEM/F-12) supplemented with 10% foetal calf serum (FCS) and penicillin (100 units/mL)/streptomycin (100ug/mL), all supplied by Invitrogen (Carlsbad, CA, USA).

### Quantitative real-time PCR

RNA was extracted using the High Pure RNA Isolation Kit from Roche (Penzberg, Germany) according to the manufacturer’s instructions. The quality, purity and amount of RNA isolated was analysed using the Nanodrop 1000 Spectrophotometer from Thermo Fischer Scientific (Waltham, MA, USA). The High Pure Paraffin Kit (Roche) was used to extract total RNA from FFPE human specimens. RNA Samples were reverse transcribed into cDNA using the Transcriptor first strand cDNA Synthesis Kit (Roche). Gene expression was measured by quantitative real time PCR (qPCR) using the 7500 Fast and Viia 7 Real-Time PCR Systems from Life Technologies (Carlsbad, CA, USA). Gene expression changes were compared relative to expression of a house keeping gene (GAPDH or β-actin) using the 2^−ΔCt^ method.

### Bisulfite sequencing

Genomic DNA was extracted from colorectal cancer cell lines using the DNeasy Blood & Tissue Kit from QIAGEN (Hilden, Germany). Genomic DNA was extracted from formalin-fixed paraffin-embedded (FFPE) tumour tissue from patients with CRC using the QIAmp DNA FFPE Tissue Kit (QIAGEN, Hilden). Extracted DNA (500–1000ng) was bisulfite treated and purified using the EpiTect Bisulfite Kit (QIAGEN)). Converted DNA was eluted in 20 μl, of which 4 μl was used to amplify 4 individual regions of the DUSP5 CpG island spanning the proximal promoter and 5′ intragenic region for PCR using Platinum Taq DNA polymerase (Invitrogen). PCR products were Sanger-sequenced by the Australian Genome Research Facility (AGRF, Melbourne, Australia). Sequences of converted DNA were evaluated using the BiQ Analyzer software^28^. Primers used were as follows: F1, 5′-TTAAATATATAGAAAAGTGGAGAAAATAGT-3′ and R1, 5′-ACCAAAACCCCAAAAAAATC-3′, to amplify a region spanning −574 to −346 nucleotides upstream of the TSS. F2, 5′-GATTTTTTTGGGGTTTTGGT-3′ and R2, 5′-TAAAACCAAATATAAATATTTCCCC-3′ to amplify a region spanning −395 to −26nt upstream of the TSS. F3, 5′-GGGAAATATTTATATTTGGTTTTATATGG-3′ and R3, 5′-CCTCCTTACGAAACATCTTAC-3′ to amplify a region spanning −49 to +309nt of the TSS. F4, 5′-GGTGGTGTTGGATTAGGGTAGT-3′ and R4, 5′-AAACCACAAAATACAAACTCCTACAA-3′ to amplify a region spanning +512 to +726 nucleotides downstream of the TSS.

### High-resolution melting methylation analysis

Bisulfite-converted genomic DNA extracted from CRC cell lines or from cores of FFPE tumour tissue from patients with CRC were subjected to HRM using the MeltDoctor HRM Master Mix (Thermo Fischer Scientific) according to the manufacturer’s instructions. A sequence spanning nucleotides −396 to −303 upstream of the TSS was analyzed. The primers used were: DUSP5-HRM-F 5′-CGGATTTTTTTGGGGTTTTGGT-3′ and DUSP5-HRM-R 5′-GATCCGACCTTCAACTTCAC-3′. The use of human CRC samples was performed in accordance with relevant guidelines and regulations of the Austin Health Human Research Ethics Committee. All patients gave written informed consent for use of their tumour material for research purposes.

### Western blotting

Total protein was extracted from cells in RIPA buffer (50 mM Tris-HCL pH 7.5, 150 mM NaCl, 1% NP-40, 0.25% sodium deoxycholate, 0.1% SDS, 5 mM EGTA). Cytosolic protein was extracted in hypotonic lysis buffer (10 mM Tris-HCL pH 7.5, 10 mM KCl, 1.5 mM MgCl_2_), following which nuclei were lysed in RIPA buffer. All buffers were supplemented with a cocktail of protease (cOmplete, Roche) and phosphatase (PhosSTOP, Roche) inhibitors. Protein concentrations were determined using the Bradford protein assay from Bio-Rad (Hercules, CA, USA). Samples were mixed with Reducing Agent and NuPAGE LDS Sample Loading Buffer (both from Invitrogen), and 20–50 µg protein resolved on NuPAGE Novex 4–12% Bis-Tris Gels (Invitrogen). Protein transfer to PVDF membranes was performed using the Invitrogen iBlot Transfer system. Antibodies used were anti-ERK1/2 (9107, Cell Signalling Technology, 1:2000), anti-pERK1/2 (4370, Cell Signalling Technology, 1:1000), anti-αTubulin (sc-8035, Santa Cruz, 1:1000), anti-DUSP5 (sc-393801, Santa Cruz, 1:1000), anti-pFRA1 (5841, Cell Signalling, 1:1000), anti-HDAC3 (AB16047, Abcam, 1:10000), anti-BRAF (sc-9002, Santa Cruz, 1:1000), anti-MYC-tag (ab9106, Abcam, 1:8000) and anti-β-actin (A5316, Sigma, 1:10000). Secondary antibodies used were fluorescent-labelled goat anti-mouse (IRdye680CW, LI-COR, 1:15000) and goat anti-rabbit (IRdye800CW, LI-COR, 1:15000). Signal was detected using the Odyssey imaging system (Li-Cor), and fluorescent intensities quantified using the Odyssey Infrared Imaging System software.

### Generation of *Vil1*-*DUSP5*^Tg^ mice

The myc-tagged coding sequence of human DUSP5 was amplified from the expression plasmid pSG5-DUSP5^8^, kindly provided by SM Keyse) using the forward primer BsiWI-DUSP5: TACGTACGGCGAATTCATGAAGGTC and the reverse primer MluI-DUSP5: TCACGCGTAGATCTGGATCCTTACAG, which introduced a BsiW I and a Mlu I restriction site at the 5′, and 3′ end of the PCR product, respectively. These sites were used to clone the PCR product into the expression vector pBSKSVillinSV40polyA (kindly provided by Sylvie Robine), which contains a 9 kb fragment of the murine villin gene promoter to generate pBSKS-*Vil1*-*DUSP5*. This construct was used for pronuclear injections (Transgenic Animal Service, University of Queensland). Transgenic founder animals were bred with C57Bl/6 mice to generate heterozygous *Vil1*-*DUSP5* ^Tg^ mice. All animal experiments were carried out in accordance with protocols approved by the Austin Health Animal Ethics Committee.

### Immunohistochemistry

Immunohistochemistry was performed on 5 μm FFPE sections. Antigen was retrieved using Dako Target Retreival Citrate buffered solution (Agilent Technologies, CA, USA), and sections incubated with anti-pERK1/2 (4370, Cell Signalling Technology, 1:100) and anti-Ki67 (MA5–14520, Thermo Fischer Scientific, 1:150) antibodies overnight at 4 °C. Sections were incubated with Dako EnVision + System HRP-conjugated anti-rabbit secondary antibody (K4011, Agilent Technologies) for 30 minutes at room temperature and subjected to chromagen-staining (3, 3′-diaminobenzidine, DAB; Agilent Technologies). Sections were counterstained with hematoxylin, scanned using a ScanScope XT system (Aperio), and analyzed using ImageScope ver.12.1.0.5029 (Aperio).

### Statistical methods

All values shown are mean ± standard error of mean. For all analyses, significance level was set at p < 0.05.

### Data availability

Additional data and reagents relating to the manuscript will be made available upon request.

## Electronic supplementary material

Supplementary Information
