# Author Correction: *DUSP5* is methylated in CIMP-high colorectal cancer but is not a major regulator of intestinal cell proliferation and tumorigenesis

**DOI:** 10.1038/s41598-023-29328-y

**Published:** 2023-02-10

**Authors:** Lars Tögel, Rebecca Nightingale, Rui Wu, Anderly C. Chüeh, Sheren Al-Obaidi, Ian Luk, Mercedes Dávalos-Salas, Fiona Chionh, Carmel Murone, Daniel D. Buchanan, Zac Chatterton, Oliver M. Sieber, Diego Arango, Niall C. Tebbutt, David Williams, Amardeep S. Dhillon, John M. Mariadason

**Affiliations:** 1 Olivia Newton-John Cancer Research Institute, Melbourne, Australia, La Trobe University School of Cancer Medicine, Melbourne, Australia; 2 Ludwig Institute for Cancer Research, Melbourne, Australia; 3 Colorectal Oncogenomics Group, Genetic Epidemiology Laboratory, Department of Pathology, The University of Melbourne, Parkville, Melbourne, Australia; 4 Systems Biology and Personalised Medicine Division, The Walter and Eliza Hall Institute of Medical Research, Melbourne, Australia; 5 Group of Biomedical Research in Digestive Tract Tumours, CIBBIMNanomedicine, Vall d’Hebron Research Institute (VHIR), Universitat Autonoma de Barcelona, Barcelona, Spain

Correction to: *Scientific Reports* 10.1038/s41598-018-20176-9, published online 29 January 2018

The Article contains an error in Figure 2, where the western blot showing tubulin has 7 lanes instead of 6 lanes.

**Figure 2:**
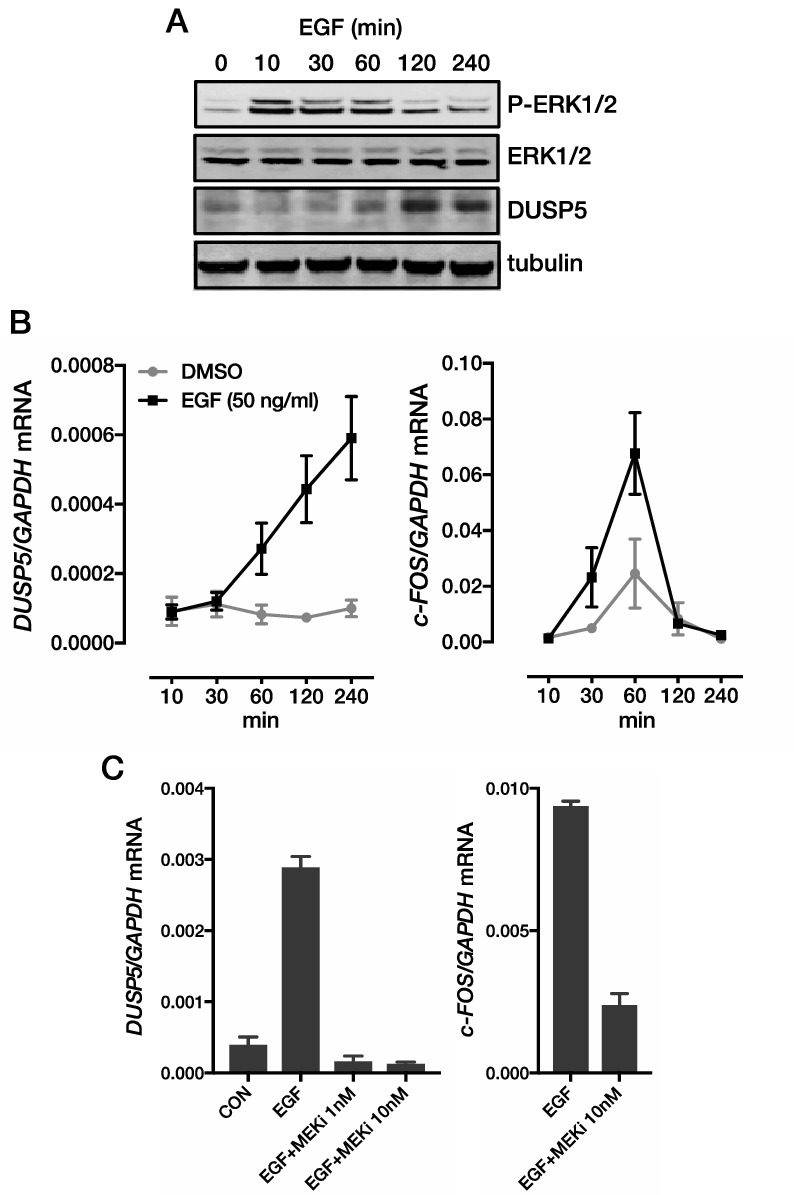
Induction of *DUSP5* by EGFR-ERK signalling in CRC cells. Time-course showing EGF-mediated induction of (**A**) p-ERK1/2 and DUSP5 protein expression, and (**B**) *DUSP5* and *c-FOS* gene expression in LIM1215 cells. (**C**) Induction of *DUSP5* and *c-FOS* mRNA by EGF requires MEK/ERK signalling. LIM1215 cells were stimulated with EGF (50 ng/ml) for 24 h in the absence or presence of the MEK inhibitor (MEKi) trametinib. *P < 0.05, **P < 0.005, *P < 0.0005.

The correct Figure 2 and accompanying legend appear below

