# Supplementary material for: DUSP5 is methylated in CIMP-high colorectal cancer but is not a major regulator of intestinal cell proliferation and tumorigenesis
Source: Sci Rep. 2018 Jan 29;8:1767. doi: 10.1038/s41598-018-20176-9 (PMC5788859; doi:10.1038/s41598-018-20176-9)

Supplementary Information

DUSP5 is methylated in CIMP-high colorectal cancer but is not a major regulator of intestinal cell proliferation and tumorigenesis

Lars Tögel, Rebecca Nightingale, Rui Wu, Anderly C Chüeh, Sheren Al-Obaidi, Ian Luk, Mercedes Dávalos, Fiona Chionh, Carmel Murone, Daniel D. Buchanan, Zac Chatterton, Oliver M Sieber, Diego Arango, Niall C. Tebbutt, David Williams, Amardeep S Dhillon and John M Mariadason

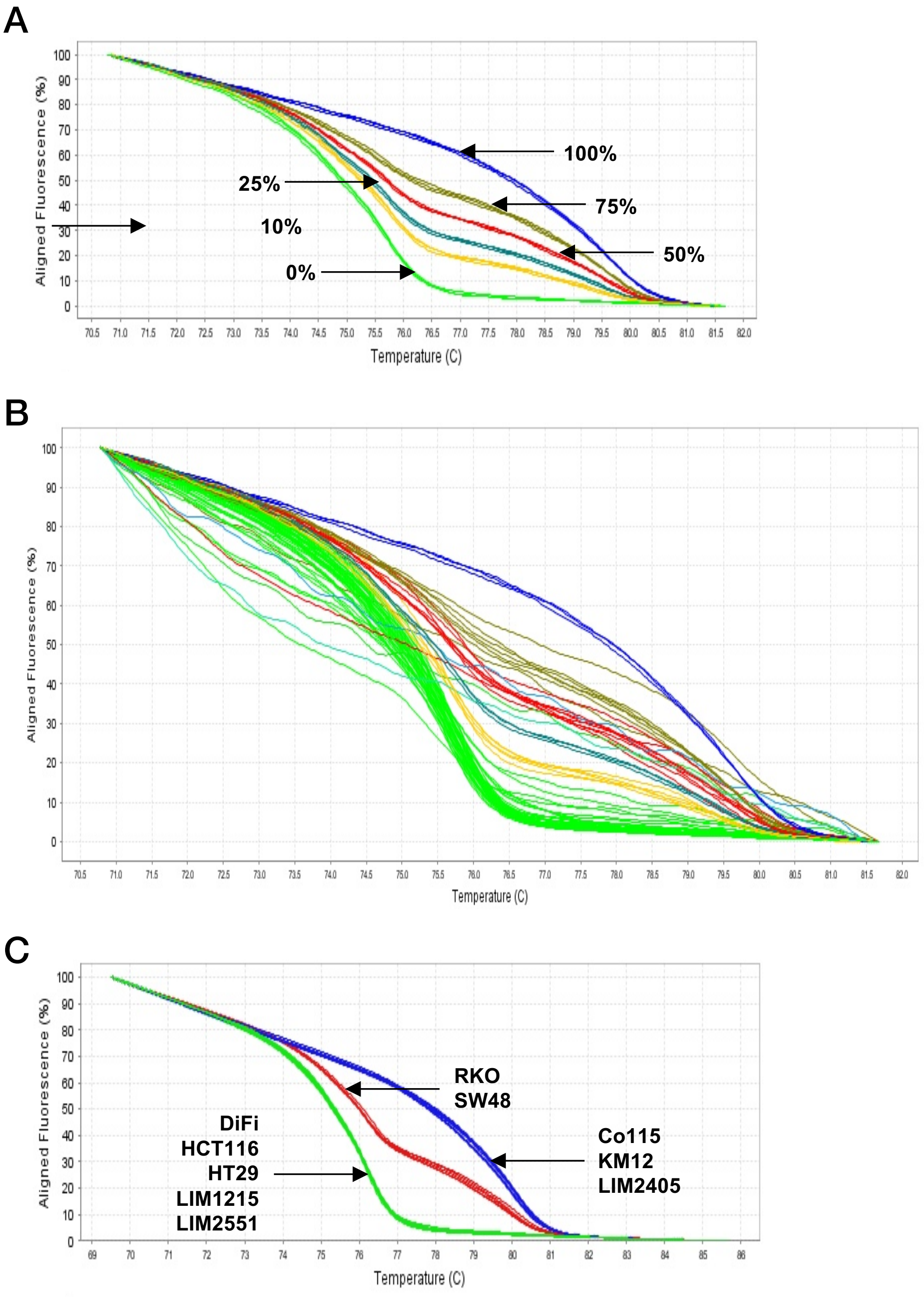

Figure S1. (**A**) Validation of HRM assay used to assess DUSP5 promoter methylation. Commercially available fully methylated DNA (100%) was diluted with unmethylated DNA to generate a standard curve which served as substrate for the assay. (**B**) Melt curves of colorectal cancer specimens. (**C**) shows the different melt curves of representative colorectal cancer cell lines with high (Co115, KM12 and LIM2405) intermediate (RKO, SW48) or low levels (DiFi, HCT116, HT29, LIM1215 and LIM2551) of DUSP5 promoter methylation as determined by bisulphite sequencing.

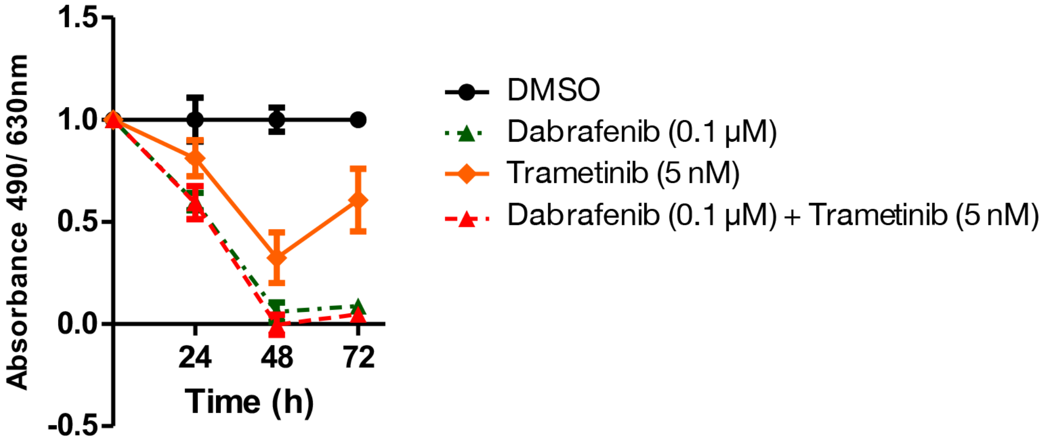

Figure S2. MTS assay assessing the effects of MEK inhibition (trametinib) alone or in combination with a BRAF inhibition (dabrafenib) on RKO cell proliferation.

Full blots: Figure 2A

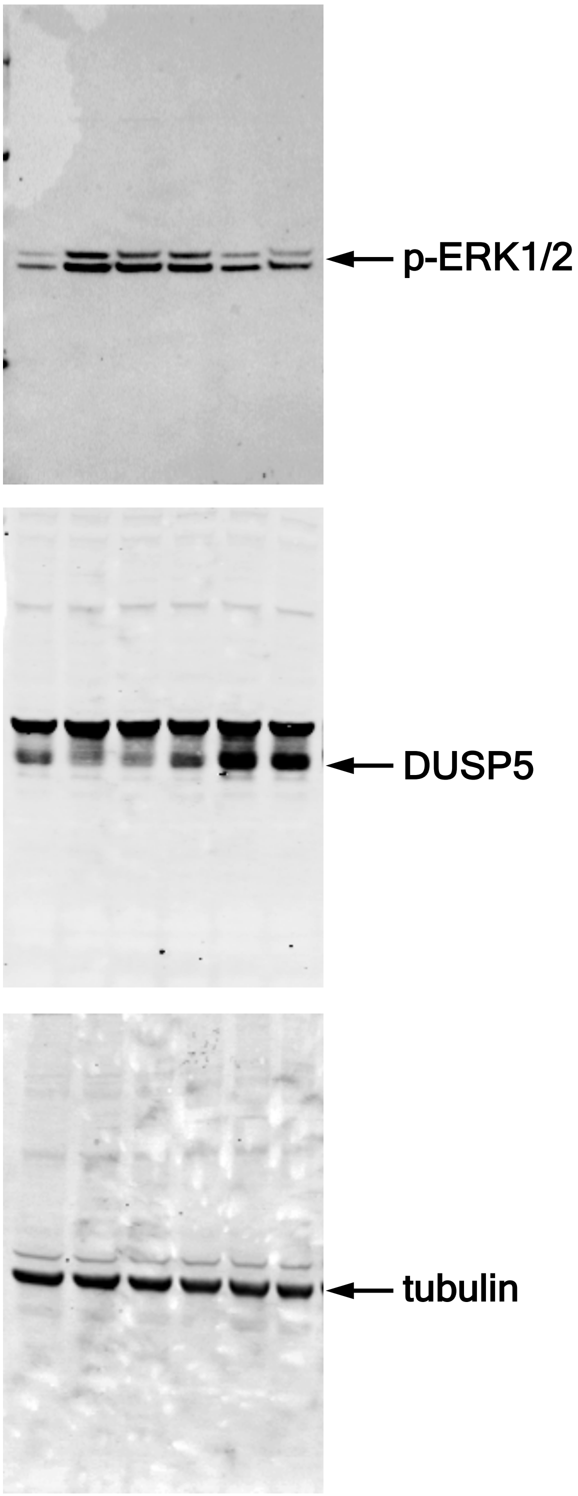

Full blots: Figure 5A

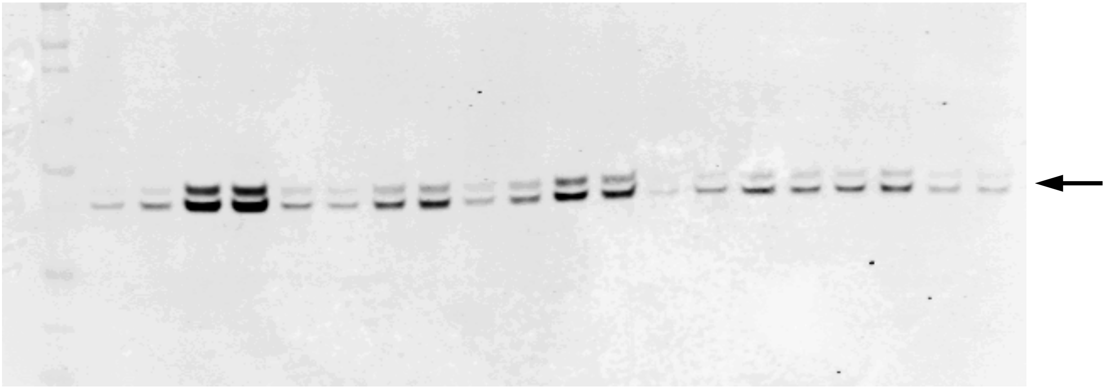

p-ERK1/2

Full blot: Figure 6A

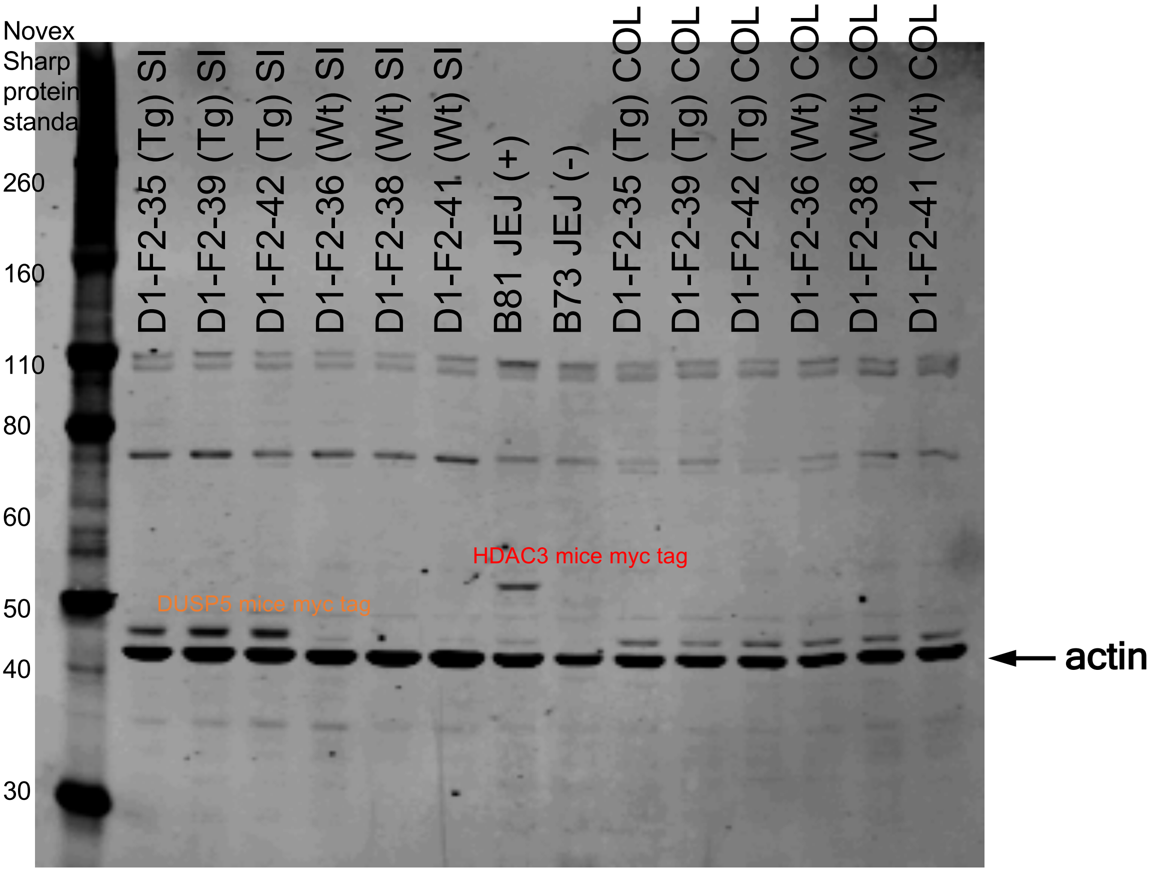

Supplement: Supplementary file 1 — Supplementary Information [file 41598_2018_20176_MOESM1_ESM.pdf]
